# Guided meditation as an adjunct to enhance postoperative recovery after cardiac surgery: study protocol for a prospective randomized controlled feasibility trial

**DOI:** 10.1186/s13063-018-3103-8

**Published:** 2019-01-11

**Authors:** Senthil Packiasabapathy, Ammu T. Susheela, Ariel Mueller, Melissa Patxot, Doris-Vanessa Gasangwa, Brian O’Gara, Shahzad Shaefi, Edward R. Marcantonio, Gloria Y. Yeh, Balachundhar Subramaniam

**Affiliations:** 1 Department of Anesthesia, Critical Care and Pain Medicine, Beth Israel Deaconess Medical Center, 330 Brookline Ave, Boston, MA 02215 USA; 2 Harvard Medical School, 25 Shattuck St, Boston, MA 02115 USA; 3 Division of General Medicine and Primary Care, Beth Israel Deaconess Medical Center, 330 Brookline Ave, Boston, MA 02215 USA; 4 Department of Anesthesia, Critical Care and Pain Medicine, Indiana University School of Medicine, 340 W 10th St #6200, Indianapolis, IN 46202 USA; 5 Beth Israel Deaconess Medical Center, 375 Longwood Avenue, W/MASCO-414, Boston, MA 02215 USA

**Keywords:** Meditation, Isha Kriya, Prehabilitation, Cardiac surgery, Postoperative cognitive decline, Delirium, Montreal Cognitive Assessment (MoCA), Depression, Anxiety, and Stress Scale (DASS-21), Pittsburgh Sleep Quality Index (PSQI), Patient-Reported Outcomes Measurement Information System (PROMIS) Surveys

## Abstract

**Background:**

Cardiac surgical procedures are associated with postoperative neurological complications such as cognitive decline and delirium, which can complicate recovery and impair quality of life. Perioperative depression and anxiety may be associated with increased mortality after cardiac surgeries. Surgical prehabilitation is an emerging concept that includes preoperative interventions to potentially reduce postoperative complications. While most current prehabilitation interventions focus on optimizing physical health, mind–body interventions are an area of growing interest. Preoperative mind–body interventions such as Isha Kriya meditation, may hold significant potential to improve postsurgical outcomes.

**Methods:**

This is a prospective, randomized controlled feasibility trial. A total of 40 adult patients undergoing cardiac surgery will be randomized to one of three study groups. Participants randomized to either of the two intervention groups will receive meditative intervention: (1) commencing two weeks before surgery; or (2) commencing only from the day after surgery. Meditative intervention will last for four weeks after the surgery in these groups. Participants in the third control group will receive the current standard of care with no meditative intervention. All participants will undergo assessments using neurocognitive, sleep, depression, anxiety, and pain questionnaires at various time points in the perioperative period. Blood samples will be collected at baseline, preoperatively, and postoperatively to assess for inflammatory biomarkers. The primary aim of this trial is to assess the feasibility of implementing a perioperative meditative intervention program. Other objectives include studying the effect of meditation on postoperative pain, sleep, psychological wellbeing, cognitive function, and delirium. These will be used to calculate effect size to design future studies.

**Discussion:**

This study serves as the first step towards understanding the feasibility of implementing a mind–body intervention as a prehabilitative intervention to improve postoperative surgical outcomes after cardiac surgery.

**Trial registration:**

Clinicaltrials.gov, NCT03198039. Registered on 23 June 2017.

**Electronic supplementary material:**

The online version of this article (10.1186/s13063-018-3103-8) contains supplementary material, which is available to authorized users.

## Background

Hospital admissions and surgeries have been increasing with time due to a shifting pattern of disease prevalence from infectious diseases to lifestyle disorders [1]. Cardiovascular diseases are increasingly seen in the elderly with various co-morbidities such as hypertension, diabetes, and renal impairment; they often require surgery [2]. Patients who undergo cardiac surgery can have significant pain, sleep impairment [3, 4], postoperative delirium (POD), and cognitive decline (POCD) [5], which can lead to increased length of hospital stay and increased healthcare expenses. Postoperative pain and sleep impairment can have a direct impact on postoperative delirium and cognitive recovery and the complications can be long lasting which can negatively impact the quality of life [6]. Evidence suggests that non-surgical preoperative patient factors such as psychological characteristics, personality traits, and preoperative cognitive impairment may also play a significant role in surgical recovery and patient satisfaction [7, 8]. Anxiety and depression in the perioperative period have been associated with increased mortality after cardiac surgery [9]. Thus, there is a definitive role for mind–body prehabilitative interventions to improve postoperative outcomes.

Surgical prehabilitation is an emerging concept that refers to interventions in the preoperative period aimed at improving postsurgical outcomes. These are pre-emptive strategies including physical therapies, pharmacological, and non-pharmacological measures to improve outcomes. In their review, Culley et al. mentioned that surgery can be compared to a fire and prehabilitation as a fire retardant [10]. Mind–body interventions include a wide range of practices and therapies that positively impact the mind’s influence on physical health [11]. Mind–body interventions have been shown to promote sleep and reduce postoperative delirium [10, 12, 13]. Integrative cardiac wellness programs can have a positive impact and can promote patient’s innate ability to heal [14].

In an expanded systematic review, it was reported that relaxation interventions and hypnosis could have a positive influence on psychological wellbeing [15]. Only two studies included in the expanded systematic review were of high quality [16, 17]. Most of the other studies did not have randomization or blinding of the outcome assessors, leading to inconclusive results. Lack of reporting pain scores was a significant limitation of the studies.

Another systematic review of randomized controlled trials examining relaxation for acute pain management similarly concluded that though there was “some weak evidence” to support the use of these therapies, the data were inconclusive, in part owing to methodological limitation [18]. The mind–body intervention was used only one day before surgery in most of the studies included in the meta-analysis. Another review provides evidence supporting the efficacy of mind–body interventions for depression, anxiety, and stress [19].

A recent study done by Kiran et al. explored the role of Rajyoga meditation, a certain type of meditation for the modulation of anxiety and serum cortisol in patients undergoing coronary artery bypass surgery. This study showed that the addition of meditation helped patients in coping with stress and they found a significant decline in anxiety levels in the postoperative period. They also found a favorable modulation of serum cortisol levels with the meditation [12]. The above studies demonstrate that breathing and meditative techniques have a biological plausibility to favorably impact outcomes. They have been shown to have established use in non-surgical chronic disease states such as fibromyalgia, cancer, hypertension, and psoriasis [20].

Finding a meditation technique that has the optimal components and that is easy enough to ensure compliance is a challenge. Meditation encompasses a family of different systems and practices including yoga meditation, mantra meditation, tai chi, mindfulness meditation, etc. Isha Kriya (IK) is a form of guided meditation that involves thought, focus, and mindful slow breathing. This regimen was chosen because it excels in simplicity and is suitable to introduce meditation to beginners.

Regarding timing and duration of application of these prehabilitative measures, research groups have studied interventions of durations in the range of 1–30 days before surgery [21, 22]. In earlier studies that examined psychosocial support, a duration as short as one day before surgery was used for intervention [18]. In preoperative exercise therapies, a 3–4-week period was used. Although optimal duration is not clearly defined, based on the current scheduling practices for surgery, two weeks is a reasonable preoperative timeframe to investigate. This randomized, controlled pilot trial will examine the feasibility of implementing a prehabilitative meditation program in the perioperative period.

## Methods / design

### Study registration

This study protocol was approved by the Committee on Clinical Investigations Institutional Review Board (IRB) at Beth Israel Deaconess Medical Center (Protocol 2017P000239). This trial was registered with the U.S. National Institutes of Health on clinicaltrials.gov with the trial identification number NCT03198039 on 23 June 2017. The trial is currently ongoing and recruiting.

### Study design

This study is a prospective, randomized, controlled, assessor blinded, single-center trial with three arms, including two experimental arms and one control arm. The study population will include adult patients undergoing coronary artery bypass grafting (CABG), valve surgeries, and/or aortic surgery at Beth Israel Deaconess Medical Center, a tertiary care hospital. Informed consent will be obtained before initiation of study procedures. Further, all trial conduct and reporting will be in accordance with components as described in the SPIRIT (Standard Protocol Items: Recommendations for Interventional Trials) checklist [23, 24].

### Aims and objectives

We hypothesized that it is feasible to implement a meditation program aimed at improving outcomes in the adult cardiac surgical population. The primary aim of this pilot study is to assess the feasibility of implementing a meditation program in the preoperative period, which will be measured in terms of adherence of the participants to the meditative regimen. Our study will further explore whether a perioperative meditation regimen can result in improvement in postoperative outcomes including pain, sleep, psychological wellbeing, cognitive function, and delirium. Given the pilot nature of the proposed trial, all secondary outcomes will be considered hypothesis generating only.

### Eligibility criteria

Patients aged ≥ 18 years, undergoing CABG, valve surgery, or isolated aortic surgery will be included in the study. To allow implementation of a prehabilitative strategy, surgeries must be scheduled at least 14 days after enrollment (Table 1).

**Table 1:** Study inclusion and exclusion criteria

Inclusion criteria	
1. Aged ≥ 18 years	
2. Undergoing any of the following types of cardiac surgery: CABG with or without valve surgery (aortic and/or mitral); isolated valve surgery; isolated aortic surgery	
3. Surgery scheduled for at least 14 days after enrollment	
Exclusion criteria	
1. Urgent and/or emergent surgery	
2. Non-English speaking	
3. Pre-existing history of psychiatric illness as documented in the medical record or divulged in history taking in pre-enrollment patient interview, such as anxiety, depression, or bipolar disorder	
4. History of cerebrovascular accident or recent history (< 3 months) of seizures	
5. History of dementia, Parkinson’s disease, Alzheimer’s disease, or other forms of cognitive decline	
6. Current use of cognition-enhancing drugs	
7. Current management for chronic pain	
8. Currently enrolled in another interventional study that could impact the primary outcome, as determined by the PI	
9. Educational attainment below high school level or equivalent	
10. Significant visual impairment	
Preoperative dropout criteria	
1. Cognitive impairment as defined by MoCA score < 10	
2. DASS-21 depression score > 10	

Patients undergoing urgent or emergent procedures or those undergoing only minimally invasive cardiac procedures will be excluded. Non-English-speaking patients and those with significant visual impairment will also be excluded, as these factors preclude performing neurocognitive assessments. Patients who do not have high-school level or the equivalent educational attainment, patients with moderate to severe cognitive impairment as defined by baseline Montreal Cognitive Assessment (MoCA) < 10 and those on cognition-enhancing drugs will be excluded. Patients with a history of cognitive impairment and psychiatric illnesses will be excluded. Patients who are actively taking pain medications will be excluded. Patients who are currently enrolled in another interventional study that could potentially impact the primary outcome will also be excluded from the study.

### Recruitment and randomization

Individuals will be identified from perioperative surgical consult lists and cardiac surgery clinic visit schedules. If a candidate is eligible for enrollment, he or she will be approached by a member of the research team to discuss the study in detail. Written informed consent will be obtained before initiation of any study procedures.

After obtaining consent, enrolled participants will be randomly assigned to one of three groups using block randomization in a 1:1:1 allocation. REDCap will be utilized to implement the randomization schema while maintaining the blind. REDCap is a secure software designed for rapid data capture tools using metadata table referenced by presentation-level operational modules [24]. Patients will be randomized to one of the following groups:

- Group 1: Patients are asked to meditate twice daily, starting from least two weeks before surgery, up until four weeks after surgery;
- Group 2: Patients are asked to meditate twice daily from the day after surgery until four weeks after surgery;
- Group 3: Patients in the control group will undergo surgery and subsequent hospital stay according to the current standard of care, which does not include meditation.

Patients who are actively meditating previously and are willing to participate in the study will be asked to refrain from meditating during the study period if they are randomized to the control group. They will be required to follow the IK meditation schedule during the period of study participation if they are randomized to one of the intervention groups.

The inclusion of a control group will allow for quantification of the natural history of surgery and postoperative complications. Further, we are utilizing two interventional groups to assess whether a prehabilitative strategy results in differences as compared to a mitigation strategy. Two intervention groups will also help us determine the optimal duration of intervention and the possible impact of duration on compliance and other secondary outcomes.

### Study intervention

IK meditation is a type of meditation that focuses on thought and mindful breathing and takes approximately 12 min to complete. This regimen was chosen because it excels in simplicity and is a great way to introduce meditation to beginners. Further, it does not incorporate any spiritual or religious focus. “Kriya” means an internal action, which is focusing on a specific thought in this case [25].

**Fig. 1:**
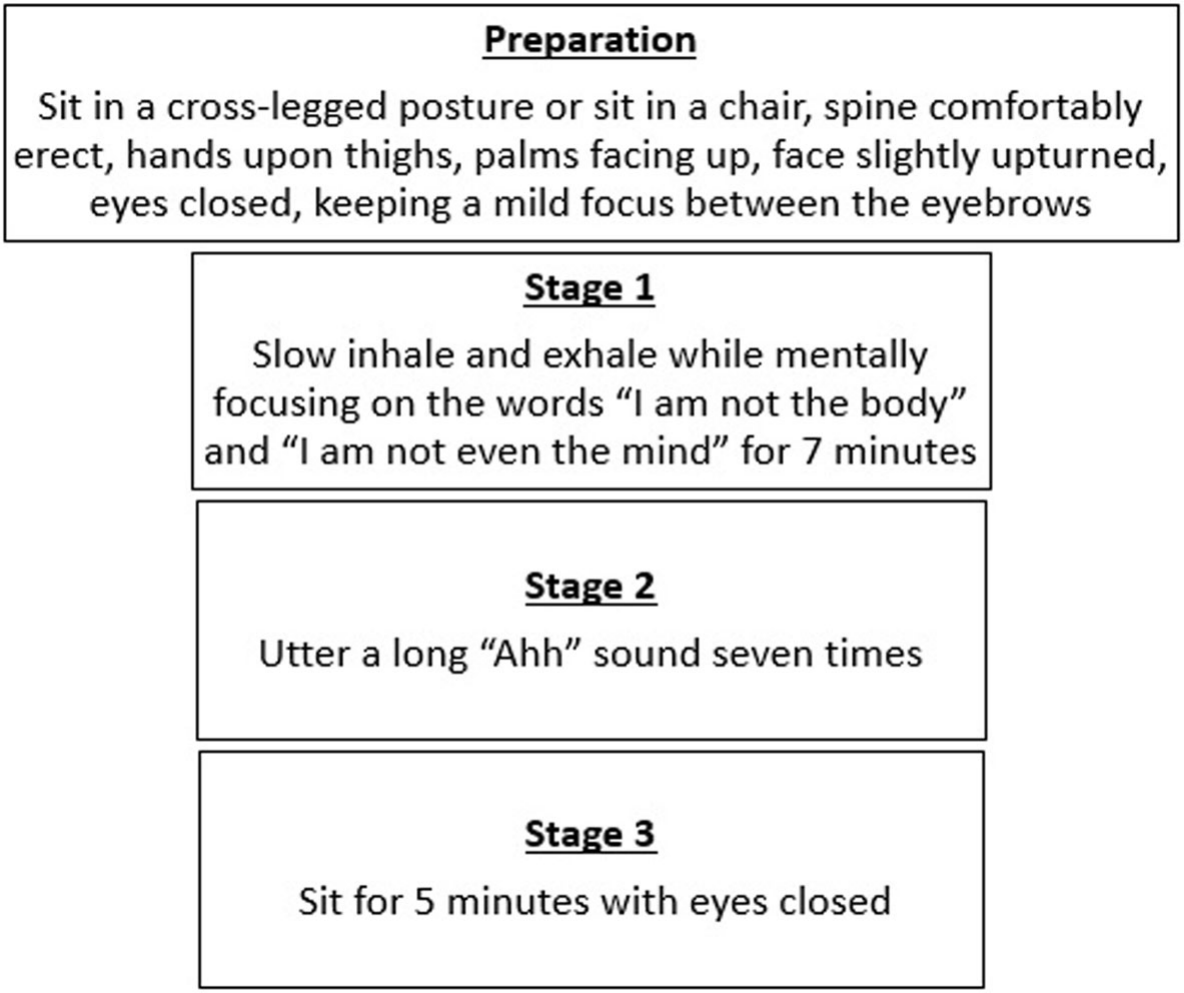
Meditation instructions. Participants in either mediation group are provided with the instructions depicted here. In short, this includes preparation and three stages in which the participant mindfully inhales and exhales slowly for 7 min, produces the sound “Ahh” seven times, and finishes with 5 min of being quiet

In brief, the participant first begins preparing for the IK practice by sitting in a cross-legged posture or sitting in a chair, spine comfortably erect, hands on their thighs, palms facing up, face slightly upturned, eyes closed, all while keeping a mild focus between the eyebrows. He or she can start meditating by slowly inhaling and exhaling while mentally focusing on the thoughts “I am not the body” during inhalation and “I am not even the mind” during exhalation. This is continued for 7 min. This is followed by uttering a long “Ahh” sound seven times. The individuals are instructed to exhale through the sound and utter the sound loud enough to feel a vibration around the mid-abdomen. The third step in the process is to sit still for 5 min with the eyes closed, as detailed in Fig. 1. This practice is to be repeated twice daily.

At the time of enrollment, individuals randomized to Groups 1 and 2 are introduced to the meditation by a trained unblinded study team member. The participant is walked through the instructions and is then allowed to practice the technique with the instructor’s guidance. Participants who are randomized to meditate only after surgery will be asked to complete the practice once to ensure they are comfortable with the instructions and to otherwise refrain from meditating until after surgery. Unblinded study team members will not conduct any other subsequent subjective study assessments to reduce any bias associated with interpreting results.

### Study outcomes

**Fig. 2:**
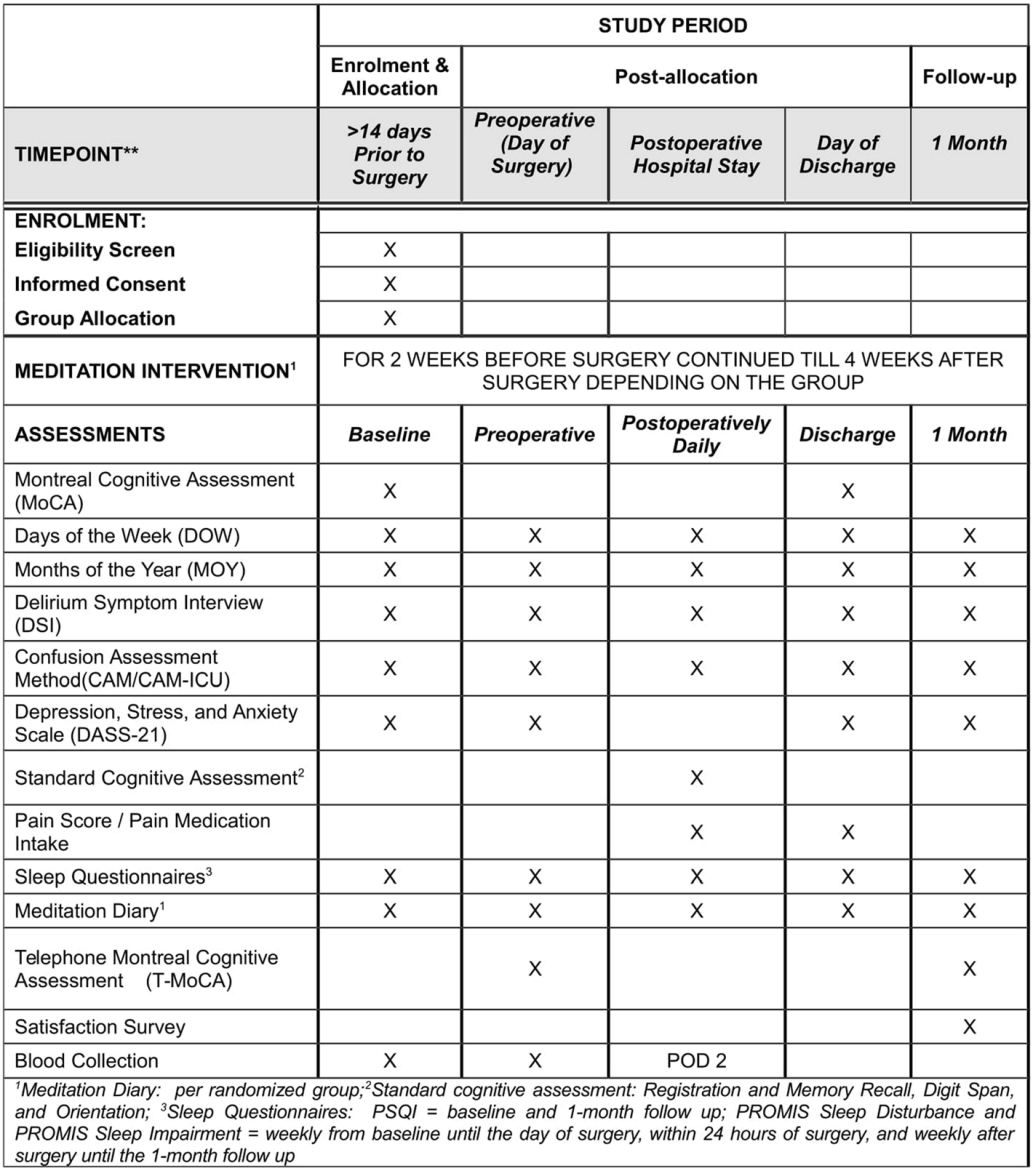
SPIRIT figure. The schedule of enrolment, interventions, and assessments in the study

The primary outcome of the study is to assess the feasibility of implementing a meditation routine in the perioperative period. This will be evaluated using the adherence data from patients’ meditation diary. Secondary exploratory outcomes include effects of the intervention on pain, sleep, psychological wellbeing, cognitive function, and delirium. A number of tools, including pain scores, questionnaires for cognition and delirium assessment, depression and anxiety questionnaire, sleep questionnaires, total opioid consumption and supplemental analgesic requirements in the first 48 h postoperatively, Intensive Care Unit (ICU) stay, and hospital length of stay, will be used to measure the secondary outcomes. In addition, we will also evaluate inflammatory biomarkers measured in plasma. All secondary outcomes will be considered hypothesis generating and interpreted as such (Fig. 2).

### Meditation adherence

To assess study feasibility, we will evaluate adherence to the study protocol. Patients in the meditation groups will be provided with meditation diaries, where they will be asked to record their daily meditation sessions. Adherence to the meditation schedule will be assessed by calculating the total number of meditation sessions completed divided by the total number of sessions required as per protocol.

### Neurocognitive and psychological assessments

A trained research team member who remains blinded to treatment allocation will perform baseline cognitive, delirium, anxiety, and depression assessments. Cognitive function will be assessed using the Montreal Cognitive Assessment (MoCA), supplemented with days of the week (DOW) and months of the year (MOY) for additional attention testing. The Delirium Symptom Interview (DSI) will be used to capture delirium. The Depression, Anxiety, and Stress scale (DASS-21) will be used to assess the baseline psychological state. Using data from the cognitive testing and DSI, the researcher will complete the Confusion Assessment Method (CAM), which includes an assessment of delirium using the CAM diagnostic algorithm. If this baseline assessment shows a DASS-21 depression score > 10 or MoCA score < 10, the individual will be excluded from the study.

To assess the changes over time, or the possible effect of perioperative meditation, a telephonic MoCA (t-MOCA) will be administered preoperatively within 24 h before surgery. These assessments will take place before initiation of any sedatives or other medications that may affect the patient’s performance or ability to complete the assessment.

On each postoperative day during the hospital stay, a blinded research team member will administer a standard cognitive assessment, including DOW, MOY, CAM, and the DSI. If an individual is intubated postoperatively, a CAM-ICU will be performed instead of a CAM assessment. If daily assessments have plateaued for three consecutive days and the patient remains CAM-negative each day, assessments will then be completed every other day if the individual remains CAM-negative, until the date of discharge. On the day of discharge, the MoCA, DOW, MOY, CAM, DSI, and the DASS-21 will be completed.

A follow-up assessment will be administered at one month (± seven days) after the date of surgery. These will be completed by a blinded research team member via telephone and will include a T-MoCA, DOW, MOY, CAM, DSI, and DASS-21. Medications that were taken 6 h before the assessments, as reported per the individual, will be documented at all time points.

### Sleep questionnaires

The Pittsburgh Sleep Quality Index (PSQI), which measures quality and patterns of sleep during the past month, will be completed at baseline and at the one-month follow-up. The PROMIS – Sleep Disturbance and the PROMIS – Sleep Related Impairment questionnaires will be completed weekly from baseline until the day of surgery, preoperatively within 24 h of surgery and weekly after surgery through the one-month follow-up. These questionnaires may be completed in person or via telephone.

### Blood collection

Blood will be collected to investigate whether the use of a meditation regimen results in a favorable modulation of inflammatory and stress biomarkers secondary to surgery. Collection will occur at baseline, preoperatively (within 24 h before surgery), and on postoperative day 2 for all participants. At each time, approximately 10 cc will be collected. All specimens will be centrifuged, plasma will be aliquoted into smaller cryovials, and frozen at − 80 °C for future use. These specimens will be stored in a biorepository which will be later used to analyze the markers of inflammation.

### Postoperative pain assessment

Postoperative pain will be assessed with a standard 11-point scale and by pain medication requirements. Pain scores will be obtained during the entire hospital stay by asking the individual and collected from the medical record when documented clinically. Pain medication intake in the first 48 h postoperatively will be ascertained from the medical record.

### Satisfaction survey

At the time of the one-month follow-up assessment, or if a participant withdraws from the study, the individuals will be asked to complete a brief satisfaction survey. This survey asks about the patients’ perception of their thinking, memory, attention, and overall wellbeing. If participants were randomized to the control group, they will be asked whether they would be interested in a meditation regime. Participants randomized to either meditation group will be asked about their experience completing the meditation practices, as well as any other feedback related to the study intervention, which will be considered during design of a future study.

### Adverse event monitoring

Since the study participants are undergoing high-risk surgery, it is expected that they may have several adverse health events during their hospital course unrelated to the study intervention. Further, the study intervention is non-invasive and carries minimal risk; therefore, we will limit the scope of our adverse event (AE) reporting to all serious AEs related to study procedures or unexpected non-serious AEs believed to be related to the study procedures. An AE includes changes in the patient’s stress and anxiety; therefore, we will monitor and report abnormal changes in the DASS-21. If the score from any of the three categories (depression, anxiety, stress) falls into the “Severe” or “Extremely Severe” range, the participant will be dropped out of the study for further clinical evaluation and management.

### Data collection and management

Data will be collected and stored on REDCap. REDCap is a web-based application that allows for customized data collection and entry [24]. The study team will build and maintain the electronic case report forms on REDCap as well as ensure data completeness and quality periodically by means of internal audits. All efforts will be made to maintain the confidentiality of patient data.

### Statistical analysis

In this feasibility study, a convenience sample of 30 participants (10 per group) will be enrolled to assess feasibility. While we expect it to be very rare, it is possible that some individuals may be determined ineligible after signing consent (because of their baseline MoCA or DASS-21 depression scores). Therefore, we plan to enroll enough patients to obtain data from 30 randomized participants. We estimate that approximately 40 patients may be enrolled to account for dropouts, loss of follow-up, and to achieve this sample size of 30. No a priori power calculation was performed.

Since the primary outcome of the study assesses the overall feasibility of conducting the trial, our descriptive study will record the proportion of patients eligible who meet inclusion without exclusion criteria and decide to participate. The statistics for overall protocol adherence to the intervention will be recorded by reporting the total number of sessions completed out of that required per protocol. We will also assess the total number of questionnaires completed out of the total required.

Descriptive statistics of data will be performed. Categorical data will be reported as proportions and assessed with the use of a chi-square or Fisher’s exact test. Continuous data will be reported as means ± standard deviations or median (quartile 1 and quartile 3) depending on distribution and assessed using analysis of variance (ANOVA). We estimate that with a small sample size it may not be acceptable to assume normality and may be appropriate to use a non-parametric equivalent such as Kruskal–Wallis test when appropriate. A trajectory analysis will be performed to assess changes in the presence of delirium or changes in cognitive function over time. Since the secondary outcomes are exploratory in nature, all tests will be two-sided, with *p* values < 0.05 considered statistically significant. Data will be analyzed using SAS 9.4 (SAS Institute Inc., Cary, NC, USA) or later. Our primary analysis will be conducted using the intention-to-treat principle. All trial reporting will adhere to the Standard Protocol Items: Recommendations for Interventional Trials (SPIRIT; Additional file 1: Table S1).

## Discussion

### Significance

Despite the growing popularity of mind–body practices, few studies have investigated their use in the perioperative period during complex surgeries such as cardiac cases. Therefore, using a comparative randomized controlled trial design, this study will lay the foundation to investigate mind–body practices in the perioperative period by exploring the feasibility of IK meditation in patients undergoing heart surgery.

### Future direction

A reasonable compliance to the meditation schedule in the intervention groups will denote feasibility and will lay down the foundation for a more elaborate study. Comparison between the two intervention groups will provide an estimate of the optimal timeframe and duration of the intervention necessary to achieve the desirable outcomes. Sensitive, quantitative tools have been used to examine and measure the magnitude of effect for various outcomes such as pain, sleep, delirium, cognitive decline, depression, anxiety, and inflammatory biomarkers. One or more positive outcomes along with their respective effect sizes will inform on the endpoints and sample size of an adequately powered study in the future.

### Limitations

One potential limitation is the possibility for loss to follow-up. This could significantly impact the study, given the relatively small sample size. A flexible timeframe for the one-month follow-up will help minimize this issue. Further, we have accounted for loss of follow-up and dropouts and have planned to enroll 40 patients in order to achieve a sample size of 30 participants. This study includes only one academic medical center and its patient population. While we are in an urban area, the study population may not be a completely representative sample of those who undergo these types of procedures. Delirium and pain may be transient and it might be difficult to measure them accurately despite the use of sensitive tools. Randomization should help minimize this limitation.

### Ethics and dissemination

This study has been approved by the Committee on Clinical Investigations, Institutional Review Board at Beth Israel Deaconess Medical Center (Protocol 2017-P-000239). Written informed consent will be obtained from all individuals before initiation of study procedures.

Results of the trial will be reported in national and international meetings, as well as in scientific journals. There are no plans to individually notify participants regarding the results of this study. If the feasibility of implementing prehabilitative meditation program in the perioperative period is proven by the study, larger studies can be undertaken to study the effect of meditation in sleep, postoperative pain and cognitive decline.

## Trial status

This trial is currently recruiting participants. Recruitment is anticipated to be completed by July 2019.

## Additional file

Additional file 1: **Table S1.** SPIRIT Checklist for the Study. (DOC 123 kb)

## Abbreviations

CABG: Coronary artery bypass graft
CAM: Confusion Assessment Method
CAM-ICU: Confusion Assessment Method – Intensive Care Unit
CARE: Center for Anesthesia Research Excellence
DASS-21: Depression, Anxiety, and Stress Scale-21
DOW: Days of the week
DSI: Delirium Symptom Interview
ICU: Intensive Care Unit
IRB: Institutional Review Board
MoCA: Montreal Cognitive Assessment
MOY: Months of the year
t-MoCA: Telephone Montreal Cognitive Assessment
